# Microbiome signatures of *Clostridioides difficile* toxin production and toxin gene presence: a shotgun metagenomic approach

**DOI:** 10.1128/msphere.00435-25

**Published:** 2025-09-25

**Authors:** Jiye Kwon, Maria A. Correa, Yong Kong, William Pelletiers, Martina Wade, Danyel Olson, Melinda M. Pettigrew

**Affiliations:** 1 Department of Epidemiology of Microbial Diseases, Yale School of Public Health 50296 , New Haven, Connecticut, USA; 2 Public Health Modeling Unit, Yale School of Public Health 50296 , New Haven, Connecticut, USA; 3 Connecticut Emerging Infections Program, Yale School of Public Health 50296 , New Haven, Connecticut, USA; 4 Department of Biostatistics, Yale School of Public Health 50296 , New Haven, Connecticut, USA; 5 Bioinformatics Resource at the W.M. Keck Foundation Biotechnology Resource Laboratory, Yale School of Medicine 12228 , New Haven, USA; 6 School of Geography and the Environment, University of Oxford, Oxford, United Kingdom; 7 Department of Environmental Health Sciences, University of Minnesota School of Public Health 43353, Minneapolis, Minnesota, USA; NC State University, Raleigh, North Carolina, USA

**Keywords:** shotgun sequencing, *Enterococcus*, antibiotics, diarrhea, *C. difficile *infection

## Abstract

*Clostridioides difficile* is an opportunistic gastrointestinal pathogen capable of asymptomatic colonization and causes diseases ranging from diarrhea to pseudomembranous colitis. Accurate diagnosis of *C. difficile* infection (CDI) is challenging and critical for treatment and control. We hypothesized that gut microbiome profiles could help distinguish *C. difficile* colonized patients with diarrhea from those with true CDI. We analyzed 172 stool samples from individuals who tested glutamate dehydrogenase positive for *C. difficile*. Participants were categorized by toxin status (i.e., toxin positive or negative) and then further classified into three toxin groups based on the production of toxin, and if not produced, whether the *C. difficile* strain carried toxin-encoding genes. We examined associations between patient characteristics, prior antibiotics exposure, microbiome community structure and function, and toxin categories. Thirty-five percent of toxin-negative participants received antibiotics despite not meeting the criteria for true CDI. *Enterococcus* species were abundant in all groups. The relative abundance of *E. faecalis* was higher among individuals with prior antibiotics exposure. Alpha and beta diversity did not differ by toxin group. After controlling for prior antibiotics exposure and previous CDI episode, the abundance of *Akkermansia muciniphila, Flavonifractor plautii*, and *Bifidobacterium adolescentis* distinguished individuals with toxin-positive *C. difficile. C. difficile* abundance did not differentiate participants with true CDI from those who were colonized. We identified associations between the gut microbiome and *C. difficile* toxin gene presence and toxin production. These results highlight the potential for microbiome-informed diagnostics to improve CDI accuracy and guide treatment decisions.

**IMPORTANCE:**

*Clostridioides difficile* colonizes humans and causes diarrhea in community and hospital settings. *C. difficile* infection (CDI) is a toxin-mediated disease, and its diagnosis is challenging. The goal of this study was to determine whether differences in the gut microbiome could help distinguish between colonized individuals and those with CDI. We examined stool samples and data from 172 individuals categorized into three groups based on the detection of toxin and, if not detected, whether toxin-encoding genes were present in the *C. difficile* strain. We identified bacteria, such as *Enterococcus faecalis*, that were more abundant in people who had used antibiotics. While the diversity of the gut microbiome did not differ by toxin group, specific gut bacteria, antibiotic resistance genes, and metabolic pathways were associated with toxin group. Our findings suggest that considering the full gut microbiome and factors like past antibiotic use could help improve the diagnosis and treatment of CDI.

## INTRODUCTION

*Clostridioides difficile* infection (CDI) is a leading cause of antibiotic-associated diarrhea and healthcare-associated infections, with an attributable cost of $6.3 billion annually in the United States (1, 2). The epidemiology of CDI is changing and is marked by increases in the incidence of community-associated infection in the past decade (1, 3). Manifestations of *C. difficile* range from asymptomatic colonization to fulminant colitis (4). Symptomatic CDI is a toxin-mediated disease, and toxins A (TcdA) and B (TcdB) are major *C. difficile* virulence factors; these toxins are cytotoxic to colon epithelial cells and can cause extensive inflammation and tissue damage (4).

Accurate diagnosis of CDI is critical for treatment, prevention, and control efforts. However, CDI diagnosis poses significant challenges (5, 6). Current tests for CDI are designed to detect *C. difficile* and/or toxin genes *tcdA* and *tcdB* (7). The reference standards for detection of toxigenic *C. difficile*, toxigenic culture, and cell cytotoxicity assay (CCA) require 24–48 hours to complete (7). Faster methods include glutamate dehydrogenase (GDH) enzyme immunoassays (EIAs), which detect the presence of *C. difficile* but cannot distinguish between toxigenic and non-toxigenic strains. EIAs targeting toxins A or B can detect *C. difficile* toxin production, but have a trade-off of low sensitivity. Diagnostic testing at many laboratories has shifted from phenotypic toxin tests (e.g., toxin EIAs) to PCR-based nucleic acid amplification tests (NAATs). NAAT-based tests can detect toxin-encoding genes; these tests have the advantage of high sensitivity but do not provide information on toxin production (5, 8). Thus, no single test can accurately diagnose CDI, and recommendations suggest two-stage testing for accurate CDI diagnosis (9).

Approximately 9% of hospitalized patients are asymptomatically colonized with *C. difficile* at the time of admission (7, 10). The high prevalence of *C. difficile* colonization among hospitalized patients, combined with the high prevalence of diarrhea, which can be due to a range of pathogens and/or non-infectious causes, poses a challenge for determining the clinical and epidemiological implications of positive *C. difficile* tests (4, 11). From the clinical perspective, inaccurate differentiation between non-toxigenic and toxigenic *C. difficile* may lead to overdiagnosis and/or overtreatment with antibiotics (5, 11). From an epidemiological and infection control perspective, it is helpful to know whether individuals are colonized with toxigenic strains that can be transmitted to others (4, 12).

The use of antibiotics is one of the main risk factors for CDI (4). Antibiotic use disrupts the gut microbiota, and subsequent microbiota dysbiosis increases an individual’s susceptibility to *C. difficile* colonization and infection (13, 14). Prior studies have examined relationships between antibiotic use, the gastrointestinal microbiota, and the risk of CDI (15, 16). However, few have utilized shotgun metagenomic sequencing to establish relationships between antibiotics, the microbiota, the antibiotic resistome, and toxin production and gene presence. We hypothesized that microbiome signatures could be used to help differentiate *C. difficile* colonized patients with diarrhea from those with true CDI. Thus, we conducted shotgun metagenomic sequencing of stool samples from individuals with diarrhea whose stool was submitted for *C. difficile* diagnostic testing. Individuals with GDH-positive *C. difficile* test results were categorized into three groups based on the detection of toxin production and, if not produced, whether toxin-encoding genes were present or absent in the *C. difficile* strain. Clinical, epidemiologic, taxonomic, and resistome data were used to identify relationships between risk factors (e.g., prior antibiotic exposure, previous CDI episode), toxin categories, and the gut microbiome.

## MATERIALS AND METHODS

### Study design and participants

**Fig 1:**
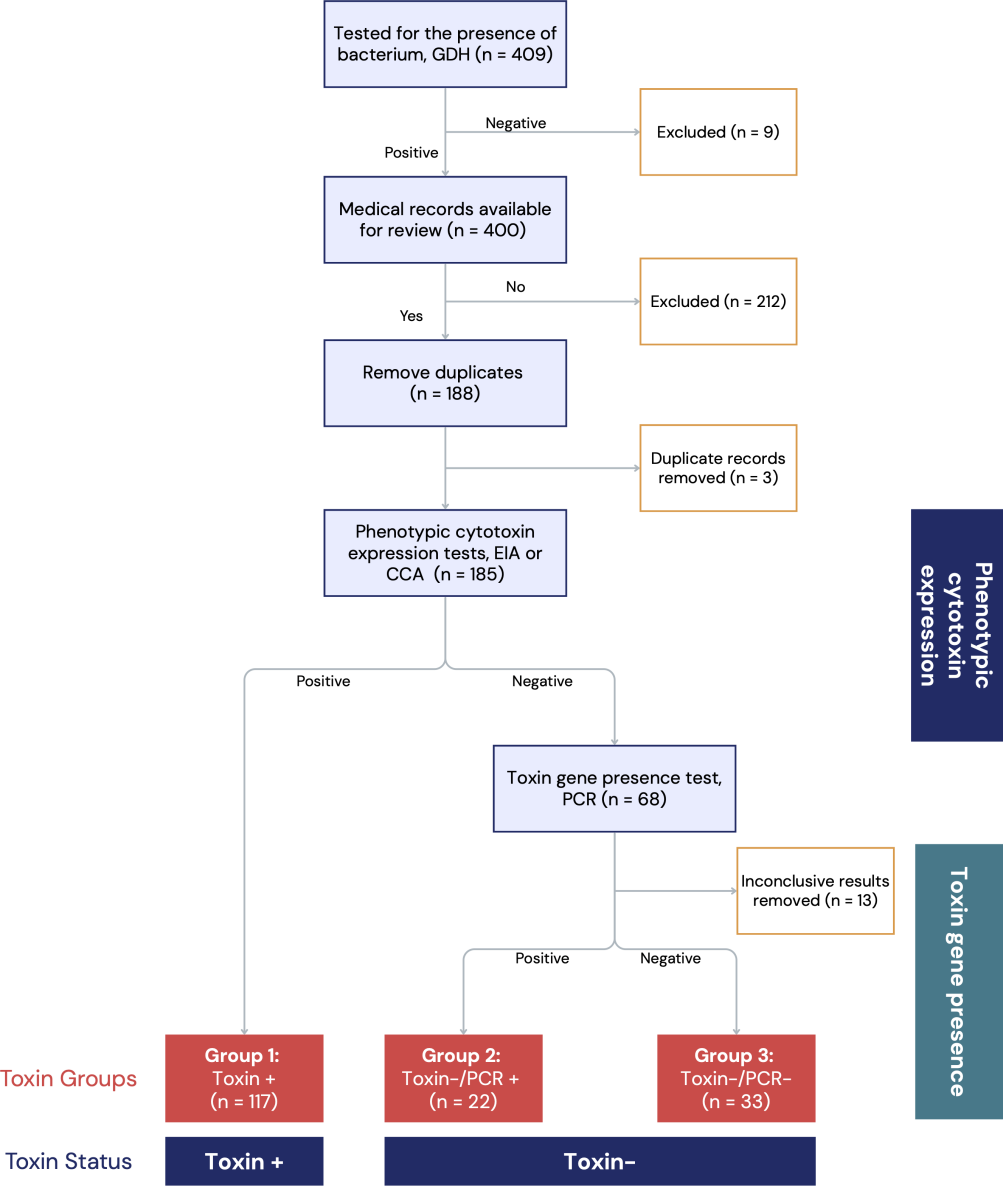
Flowchart diagram summarizing toxin categories. Toxin status is defined as toxin positive or negative and toxin groups: group 1 Toxin+, representing individuals with a test positive for toxin production; group 2 Toxin−-/PCR+, representing no toxin production and toxin gene present; and group 3 Toxin-−/PCR-−, representing samples from individuals colonized with a *C. difficile* strain that did not carry toxin A or B genes. Purple boxes represent tests conducted. Red and blue boxes represent three toxin groups and toxin status as defined in the study, respectively. All patients exhibited diarrheal symptoms prior to testing for the presence of the bacterium.

Yale University’s institutional review board approved this study with a waiver of consent under protocol 2000026568. Stool samples were obtained through a collaboration with Connecticut’s Emerging Infections Program, which conducts *C. difficile* surveillance in Connecticut (17). Information on testing algorithms and participant toxin group assignments is summarized in Fig. 1. From April 26, 2019, to October 18, 2020, a total of 409 residual stool samples were obtained from individuals with diarrhea symptoms who tested GDH positive for *C. difficile* at a Connecticut hospital. Phenotypic test results for cytotoxin production (EIA or CCA) were available, and medical chart reviews were conducted for 188 samples. Of these, three individuals were identified with >1 stool sample and had their subsequent samples removed from the study (*n* = 185). Data extracted from the medical charts included past medical history, use of antibiotics, proton pump inhibitors, histamine type 2 receptor antagonists (H2 blockers), and immunosuppressants, as well as details on CDI treatment and recent contact with healthcare.

### *C. difficile* diagnostic testing, toxin status, and toxin group classification

Participants with GDH-positive *C. difficile* were classified by cytotoxin status based on a cytotoxin *C. difficile* diagnostic testing algorithm (9). Cytotoxin production results were based on one of two phenotypic assays, EIA or CCA. Participants whose test results were GDH+ and toxin positive, based on any of the phenotypic toxin assays, were classified as positive for toxin production (Toxin+). Participants whose tests were GDH+ and toxin negative were classified as toxin production negative (Toxin-−). Samples that were Toxin- −(*n* = 68) were further subjected to PCR in a research laboratory to determine whether the *C. difficile* strains carried toxin-encoding genes *tcdA* or *tcdB* as described under the section below on fecal specimen processing. Samples that were PCR positive for *tcdA* or *tcdB* were classified as Toxin-−/PCR+, and those that were *tcdA* and *tcdB* PCR negative were classified as Toxin-−/PCR-−. Thus, there were two “toxin categories” for analyses. Toxin status, defined as Toxin+ or Toxin−, and toxin groups, defined as follows: group 1 Toxin+, representing individuals with a test positive for toxin production; group 2 Toxin-−/PCR+, representing no toxin production and toxin gene present; and group 3 Toxin-−/PCR-−, representing samples from individuals colonized with a *C. difficile* strain that did not carry toxin A or B genes.

### Fecal specimen processing, PCR, and shotgun metagenomic sequencing

Stool samples were collected from the hospital laboratory and stored at −30°C until use. Sixty-eight stool Toxin-− samples were cultured for *C. difficile* and subjected to PCR for the detection of toxin-encoding genes. *C. difficile* was isolated and cultured from stool samples using cycloserine cefoxitin fructose agar with horse blood and taurocholate (CCFA-HT) selective media plates (Anaerobe Systems, Morgan Hill, CA) and brain heart infusion broth (Becton Dickinson, Sparks, MD) (37 g), agar (14 g), defibrinated horse blood (7%) (Quad Five, Ryegate, MT), L-Cysteine HCl (500 µg/mL) (Calbiochem) non-selective media plates were used to sub-culture isolates. Plates were placed in the anaerobic chamber at 37℃ for 48 hours. Samples were prepared for PCR using a boil lysis method and subjected to PCR using methods described by Persson et al. (18).

 For shotgun metagenomic sequencing, stool samples were thawed, and the DNA was extracted using the PureLink Microbiome Purification Kit (Invitrogen). After DNA quantification using the Quant-iT Assay Kit (Invitrogen), samples were prepared for sequencing using the NEBNext Ultra II FS DNA Library Prep Kit for Illumina (New England Biolabs). Shotgun metagenomic sequencing was carried out on an Illumina NovaSeq at the Yale Center for Genome Analysis. Of the 185 GDH-positive samples from unique individuals with corresponding medical record data (see Fig. 1), there were 13 stool samples with inconclusive PCR results and zero metagenomic *C. difficile* reads; these samples were removed from the analysis. Thus, the final data set for this study consisted of stool samples and clinical and epidemiologic data from 172 individuals.

### Bioinformatic and statistical analyses

Btrim software was used to trim and filter out low-quality shotgun metagenomic sequence reads (19). We obtained a median (range) of 27,300,656 (10,751,879–48,186,574) trimmed pairs from our 172 samples. The metagenomic shotgun sequencing data were further processed using the bioBakery 3 tools (20). Microbial species-level taxonomic profiling and the relative abundance of microbial species were calculated using MetaPhlAn 3.0 (20). For each sample, relative abundances sum up to 1, representing 100%. The HUMAnN 3.0 pipeline was used for functional profiling of the microbial community from shotgun metagenomic sequences (20). The antibiotic resistance gene (ARG) online analysis pipeline with the expanded structured ARG (SARG) database v2.0 was used for classification and quantification of ARGs (21). ARG data were normalized using a reference set of essential single-copy genes, and data are presented as resistance genes per prokaryotic cell at either the type (i.e., antibiotic class) or subtype level (i.e., individual gene level).

Metagenomic, clinical, and demographic data were imported into R for statistical, community composition, and differential abundance (DA) analyses (22). Prior antibiotic exposure was defined as any antimicrobial therapy taken in the 12 weeks prior to the date of stool collection. For this study, a case was defined as an incident if the individual did not have a prior CDI in the 8 weeks prior to the date of stool collection. Participants were classified as having a previous CDI episode if medical chart review revealed a CDI episode more than 8 weeks before the date of incident *C. difficile* stool collection. Recurrent CDI was defined as having CDI episodes ≥ 2 weeks but ≤8 weeks since *C. difficile* stool collection among individuals with samples positive for cytotoxin production (i.e., Toxin+) (17).

The relationship between any prior antibiotics exposure and prior antibiotics by class on differential phenotypic cytotoxin detection was explored via univariate logistic regression. Prior to any microbiome data analysis, we identified one outlier whose microbiota composition consisted solely of 100% *Gardnerella vaginalis* and excluded the sample in subsequent ARG, taxonomic, and pathway profiling analyses. We compared both alpha and beta diversity across toxin groups. Shannon diversity (natural log) was calculated as a measure of alpha diversity for each stool sample (23, 24). We used the non-parametric Kruskal-Wallis and Wilcoxon test to compare the median alpha diversity between groups, as appropriate. We used the Bray-Curtis dissimilarity index as the metric for beta diversity, as it has been shown to be able to detect differences between groups with high sensitivity (25, 26). For beta-diversity visualization, we used non-metric multidimensional scaling to condense complex multidimensional microbiome data into a low-dimensional space (27, 28). We further supplemented the analysis of between-group differences in beta diversity using a more robust method, permutational analysis of variance (PERMANOVA) (25, 29, 30) via the adonis2 function in the vegan R package with 4,000 permutations (31), to test whether the overall gut microbiota composition varied significantly by toxin groups. First, we tested the null hypothesis that the centroids or group means were equivalent overall across all groups being tested. Next, we performed a post hoc pairwise comparison to test and determine which pairs might be different from each other while correcting for multiple comparisons.

For the identification of key taxa associated with the outcome of interest, we conducted DA analysis using the microbiome multivariable associations with linear models 2 (MaAsLin2) package (32). MaAsLin2 was selected based on its ability to preserve statistical power while sufficiently controlling for the false discovery rate (FDR) even in the presence of multiple covariates and repeated measures (32). We uniformly removed all-zero features across taxonomic, ARG, and metabolic pathway data sets. For taxonomic and ARG data, we applied a minimum prevalence filter of 10% and excluded features with a low variance (<0.001) or with a mean relative abundance below 1% as part of MaAsLin2 analyses. For metabolic pathway data, we applied a more stringent prevalence filtering of 20% to reduce data noise and enhance data quality (33), due to a high number of features with extremely low variance. The significance level for DA analysis was set to a *q*-value of <0.25 after the *P*-values were FDR-adjusted (BH, *q*-values). A summary of the MaAsLin2 equations used in each analysis is in Supplementary methods S1.

## RESULTS

### Participant characteristics and toxin group

The characteristics of 172 participants, categorized by toxin group, are shown in Table 1. Sixty-eight percent had positive diagnostic test results indicative of true CDI (i.e., group 1 Toxin+; *n* = 117). The remaining participants were divided based on PCR-based toxin gene presence; 22 participants had Toxin−-/PCR+ tests and were assigned to group 2, and 33 individuals had Toxin−-/PCR-− tests and were assigned to group 3. The demographic characteristics of the participants, including age, sex, race, and/or ethnicity, did not significantly differ by toxin group (Table 1). A majority of the participants, 83%, had exposure to healthcare. However, healthcare exposure did not significantly differ by toxin group (Table 1). Twelve percent of participants with Toxin+ samples had recurrent CDI; by definition, there were no participants with recurrent CDI who had Toxin−-/PCR+ or Toxin−-/PCR− samples (Table 1).

**TABLE 1:** Patient cohort characteristics by toxin groups^*a*^

Characteristic	Group 1: Toxin+ (*N* = 117)	Group 2: Toxin−/PCR+ (*N* = 22)	Group 3: Toxin−/PCR-(*N* = 33)	*P* Value^*b*^
Age (years)				0.52
Median [IQR]	66.0 [53, 75]	63.5 [43, 75]	63.0 [49, 69]	
Sex				0.80
Male	59 (50)	10 (46)	18 (55)	
Female	58 (50)	12 (55)	15 (46)	
Race				0.16
Asian	1 (1)	0 (0)	0 (0)	
Black	12 (10)	4 (18)	10 (30)	
White	93 (80)	15 (68)	21 (64)	
Unknown	11 (9)	3 (14)	2 (6)	
Ethnicity				0.97
Non-Hispanic or Latino	104 (89)	20 (91)	30 (91)	
Hispanic or Latino	12 (10)	2 (9)	3 (9)	
Not reported	1 (1)	0 (0)	0 (0)	
Case definition				0.05
Hospital-acquired	61 (52)	18 (82)	23 (70)	
Healthcare-associated	34 (29)	1 (5)	6 (18)	
Community-acquired	22 (19)	3 (14)	4 (12)	
Prior antibiotics exposure				0.02
Yes	71 (61)	8 (34)	13 (39)	
No	46 (39)	14 (64)	20 (61)	
Antibiotic treatment for incident CDI				< 0.001
Yes	115 (98)	10 (46)	9 (27)	
No	2 (2)	12 (55)	24 (73)	
Antibiotic (first line)				< 0.001
Metronidazole	2 (2)	1 (5)	0 (0)	
Vancomycin	109 (93)	8 (34)	7 (21)	
Other	4 (3)	1 (5)	2 (6)	
No antibiotic treatment	2 (2)	12 (55)	24 (73)	
Previous CDI episode**^*c*^**				< 0.001
Yes	12 (10)	8 (34)	16 (49)	
No	104 (89)	14 (64)	17 (52)	
Recurrent CDI				0.03
Yes	14 (12)	0 (0)	0 (0)	
No	103 (88)	22 (100)	33 (100)	

^*a*^ CDI: *Clostridioides difficile* infection.

^*b*^ Toxin: phenotypic detection of cytotoxin production.

^*c*^ CDI episode unknown (1).

Participants differed by toxin group in clinical characteristics such as prior antibiotics exposure, antibiotic treatment for CDI, type of antibiotic for treatment, and previous CDI episode. Notably, the Toxin+ group had a significantly higher proportion of participants with prior exposure to antibiotics and treatment for incident CDI (Table 1). Of concern, a considerable proportion of participants, whose samples were negative for toxin production (Toxin−-/PCR+) or toxin genes (Toxin−-/PCR−-), also received antibiotic treatment for CDI, 46% and 27%, respectively.

The relative risk of CDI has been shown to vary by the type of antibiotic exposure (34). Thus, we sought to examine relationships between antibiotics and toxin status (i.e., Toxin+ vs. Toxin- −samples). Any prior antibiotics exposure was associated with a 2.50 (95% confidence interval [CI]: 1.30, 4.88) higher odds of Toxin+ status (Table S1). Having prior exposure to beta-lactam, cephalosporin, or fluoroquinolone antibiotics was also significantly associated with higher odds of Toxin+ status (Table S1). While vancomycin exposure was most common among all antibiotic classes, prior exposure alone was not significantly associated with positive toxin status.

### Taxonomic profile of stool samples

**Fig 2:**
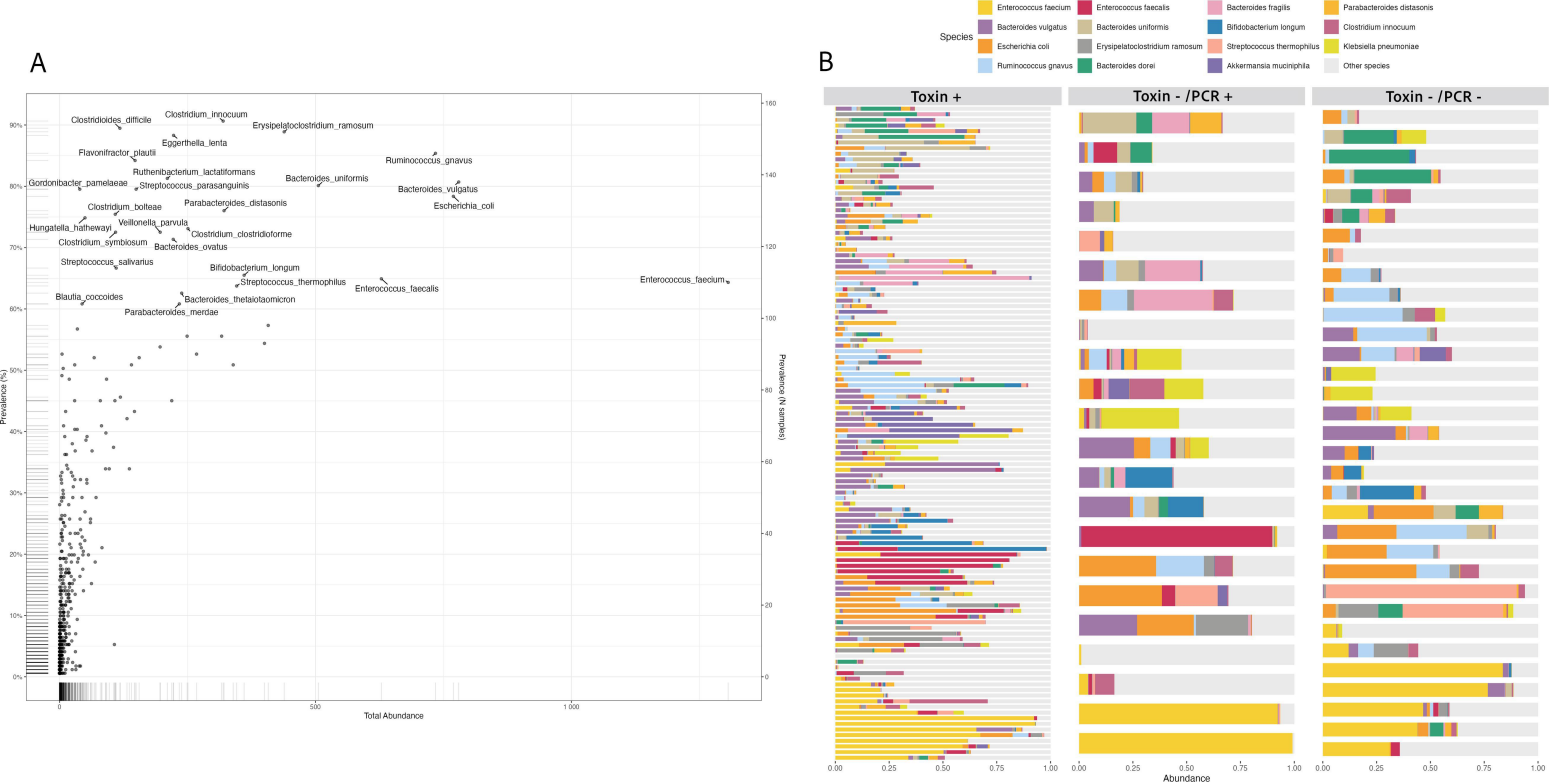
Overview of microbiome composition across samples. (**A**) Scatter plot showing total abundance (X-axis) versus prevalence (Y-axis) of microbial taxa across all samples (*n* = 171). Species present in ≥60% of samples are labeled as key taxa. (**B**) Stacked bar plots of species by toxin groups: Toxin+ (*n* = 116), Toxin−/PCR+ (*n* = 22), and Toxin−/PCR− (*n* = 33). Each bar corresponds to an individual sample, and the top 15 most abundant species are annotated with colored bars.

A total of 633 unique bacterial taxa were identified in GDH-positive stool samples from participants. A scatter plot highlighting key taxa (species present in ≥60% of samples) showed that *Enterococcus faecium* and *Enterococcus faecalis* were among the most abundant species identified across all study participants, regardless of their toxin status or toxin group (Fig. 2A). Among the 27 key taxa identified, we also investigated species-level correlation across each species (Fig. S1). We observed no obvious clustering of species distribution by toxin group (Fig. 2B). Other taxa with high overall relative abundance included *Bacteroides* species*, Escherichia coli, Ruminococcus gnavus, Streptococcus thermophilus,* and *Akkermansia muciniphila* (Fig. 2B).

### Microbial community structure across *C. difficile* toxin groups

**Fig 3:**
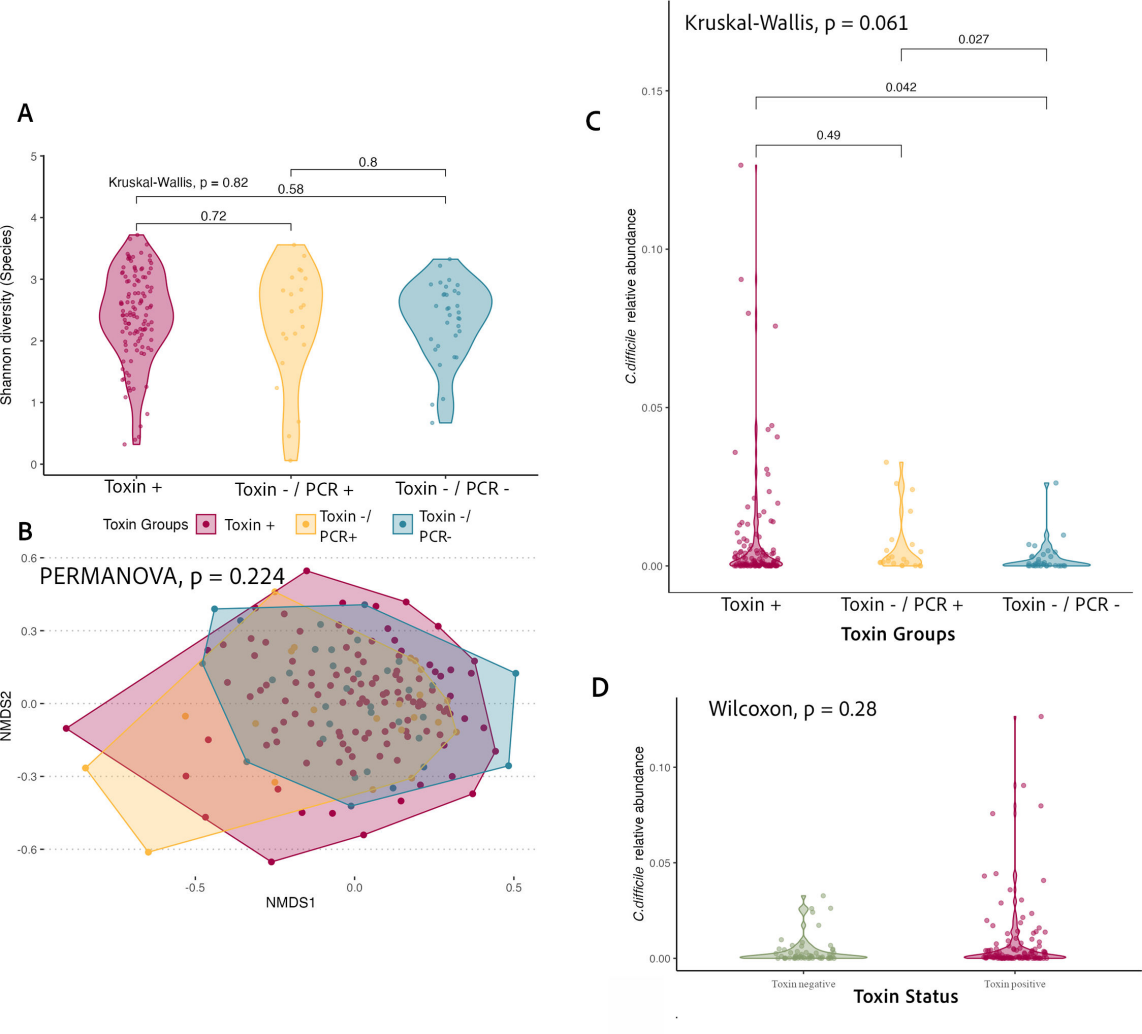
Microbial diversity and *C. difficile* relative abundance across toxin groups and clinical outcomes. (**A**) Alpha diversity (Shannon index) of the gut microbiota stratified by ***C.*** *difficile* toxin group. (**B**) Beta-diversity ordination plot showing microbial community composition across toxin groups. No significant differences in overall gut microbiota composition across groups (*P* = 0.22; PERMANOVA; 0.33 variation in dispersion). (**C**) Relative abundance of *C. difficile* across toxin groups. (**D**) Relative abundance of *C. difficile* stratified by toxin status. Pairwise comparisons were conducted using the Wilcoxon rank-sum test, with corresponding *P*-values displayed above bars. Overall group differences were assessed using the Kruskal–Wallis test.

We compared alpha diversity (i.e., Shannon diversity) across toxin groups (Fig. 3A); no significant differences were observed in alpha diversity across the three groups (Kruskal-Wallis test, *P* = 0.82). Next, we examined beta diversity across the toxin groups. An ordination plot was created to visualize the microbiome information of all samples relative to other samples (Fig. 3B). We did not identify significant differences in the compositional profile of the gut microbiota by toxin group, either before or after adjusting for prior antibiotics exposure and case definition (adjusted *P* = 0.22; PERMANOVA; Table S2). Similarly, pairwise comparisons revealed no differences (Table S3). In the adjusted model, we also examined the effects of additional covariates, prior antibiotics exposure, and case definition (hospital-acquired, healthcare-associated, or community-acquired) on beta diversity. PERMANOVA results revealed beta-diversity differences across both variables (case definition *P* < 0.001; prior antibiotics exposure *P* = 0.004, Table S2). However, the differences may be an artifact of wide variances and not true compositional differences (*P* < 0.001 and *P* = 0.007 variation in dispersion, respectively). Therefore, caution is warranted when interpreting these results.

The relative abundance of *C. difficile* in stool samples is higher in individuals with CDI compared to those who are colonized (35). To further understand *C. difficile* abundance across different factors, we compared its relative abundance across toxin groups and CDI recurrence among Toxin+ samples. Pairwise comparisons across the toxin groups indicate that participants colonized by non-toxigenic strains (i.e., Toxin−-/PCR-−) tended to have the lowest relative abundance of *C. difficile* (Fig. 3C). The relative abundance of *C. difficile* was not significantly different by toxin status (Fig. 3D) nor among those who experienced CDI recurrence vs did not (Wilcoxon, *P* = 0.38).

### Risk factors: impact of prior antibiotics and previous CDI on the microbiome

**Fig 4:**
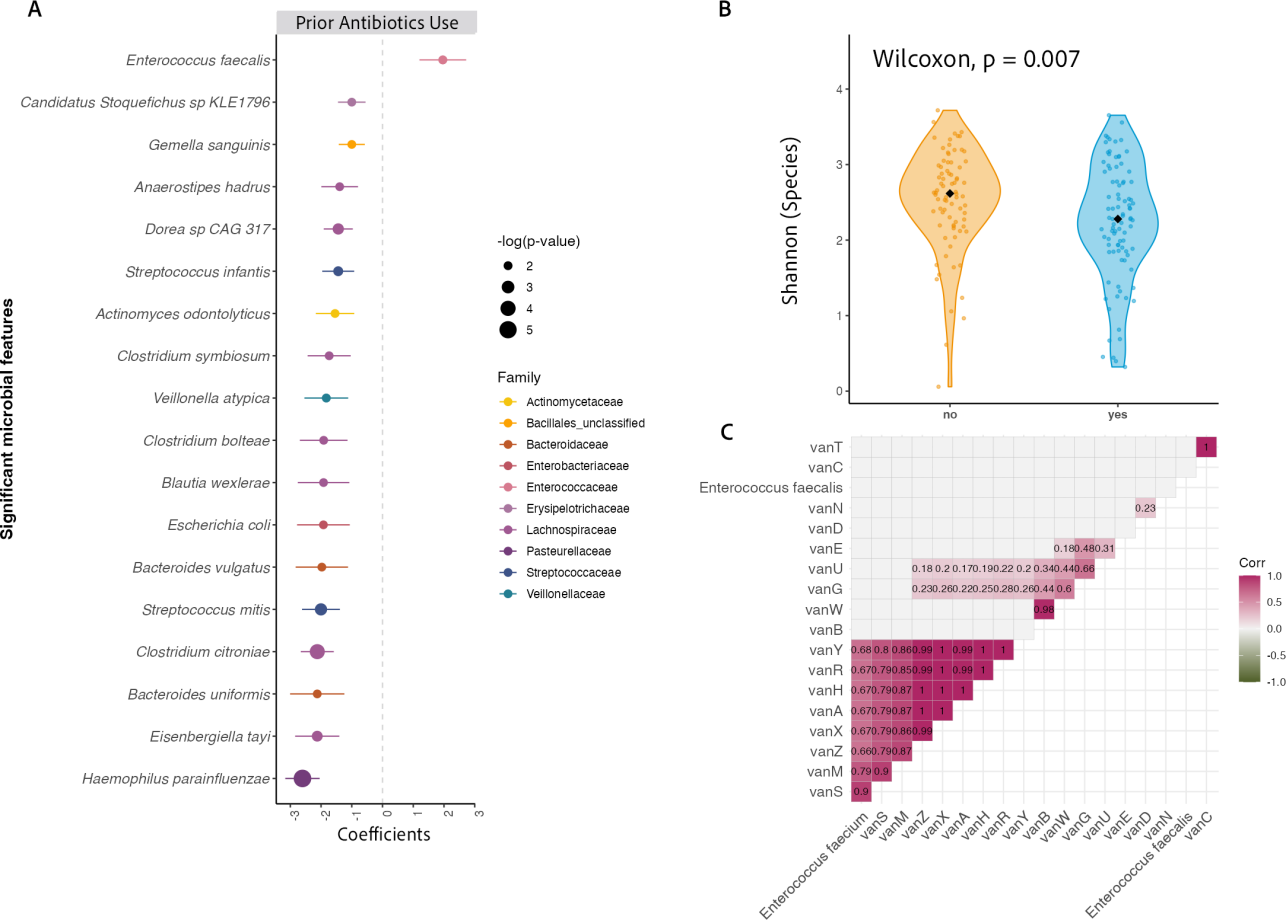
Impact of prior antibiotics exposure on microbial composition, diversity, and ARG associations. (**A**) DA analysis identifying microbial taxa significantly altered by prior antibiotics exposure. (**B**) Alpha diversity (Shannon index) of the gut microbiome comparing individuals with and without prior antibiotics exposure. (**C**) Correlation between *Enterococcus* species abundance and vancomycin-associated ARG elements.

Antibiotics are a primary risk factor for CDI, and prior antibiotics exposure differed across toxin groups. We conducted a DA analysis to identify individual species associated with differences in prior antibiotics exposure. Higher relative abundance of *E. faecalis* distinguished individuals with prior antibiotics exposure compared to those who did not have prior antibiotics exposure (Fig. 4A). The relative abundance of 17 different species was significantly higher in individuals who did not have exposure to prior antibiotics compared to those who did (Fig. 4A). Concurrently, participants without prior antibiotics exposure had higher alpha diversity than those who were previously exposed to antibiotics (Fig. 4B).

Given the abundance of *Enterococcus* species in all our GDH+ samples, particularly among those who had prior antibiotics exposure, we investigated correlations with vancomycin-associated ARG subtypes. We found a strong positive correlation between *E. faecium* and several vancomycin ARGs, notably *vanA* (Fig. 4C). Additionally, clusters of genes including *vanS, vanH, vanX,* and *vanR* were highly correlated with *E. faecium* abundance (Fig. 4C). At the antibiotic class level, differences in vancomycin ARGs were not evident, but the abundance of fosmidomycin and kasugamycin was markedly higher among those without prior antibiotics exposure (Fig. S2).

The proportion of participants with a previous CDI episode differed across groups (Table 1). In addition to species-level DA analysis by antibiotic exposure (Fig. 4A), comparison between previous CDI episode (yes/no) showed higher relative abundances of *E. coli* in individuals who experienced a previous CDI episode (Fig. S3).

### Species-level comparisons: previous CDI episode, prior antibiotics exposure, the gut microbiome, and toxin categories

We hypothesized that resolution at the individual species level might be needed to identify differences by known risk factors and across different toxin categories. First, we conducted multiple univariate species-level DA analyses to identify microbial taxa predictive of toxin group or toxin status. There were no taxa identified as discriminant species for toxin status or any of the toxin groups after correcting for multiple comparisons via FDR adjustment (data not shown, q < 0.25 as cut-off).

**Fig 5:**
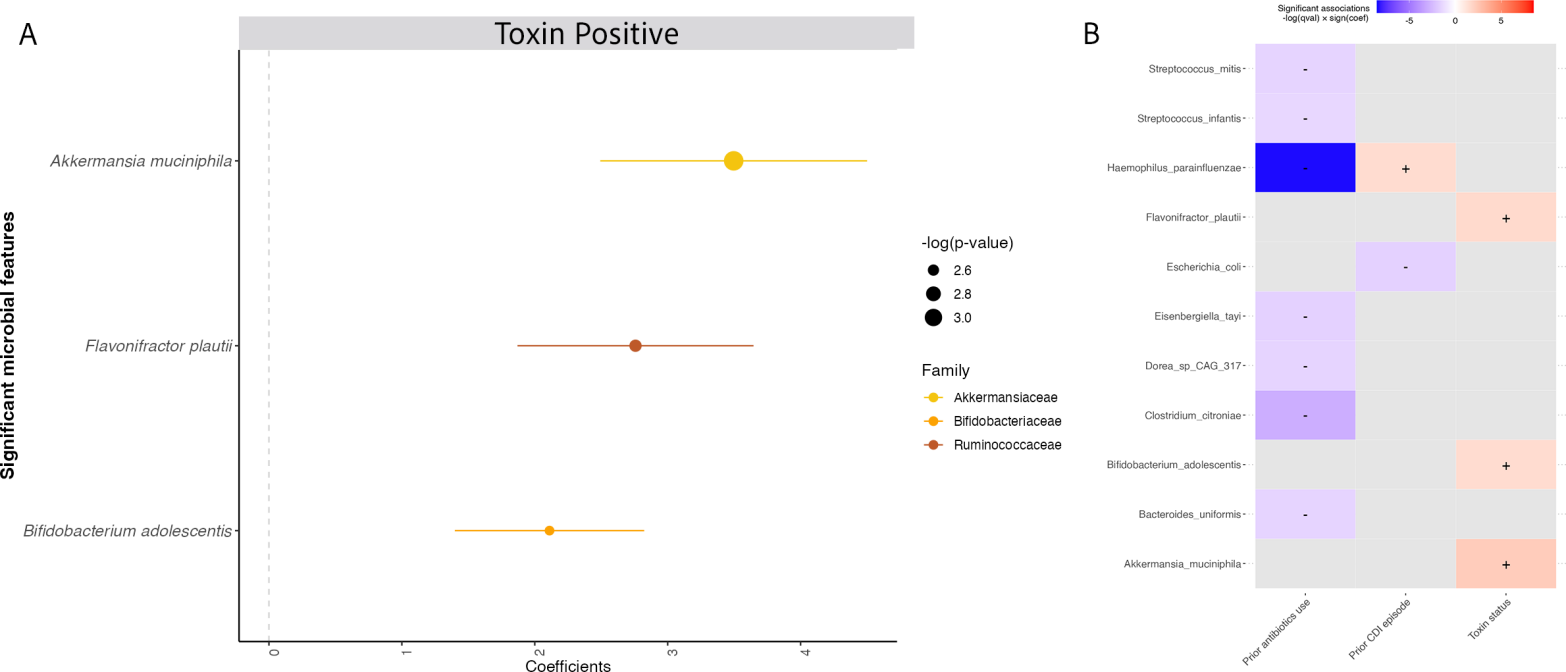
Significant microbial taxa associated with toxin status adjusting for prior antibiotics exposure and previous CDI episode. (**A**) A positive coefficient is associated with a positive Toxin+ status, and a negative coefficient is associated with Toxin– status. Colors indicate the taxonomic family of the microbial feature, and the size of the circle corresponds to the magnitude of statistical significance. Features with a *q*-value of <0.25 were plotted. (**B**) Heatmap displaying associations between taxa and additional covariates included in the model (e.g., prior antibiotics exposure, previous CDI episode).

We conducted a multivariable DA analysis to disentangle relationships between prior antibiotics exposure, previous CDI episode, taxa, and toxin status. Thus, we adjusted for both prior antibiotics exposure status and previous CDI episode as potential confounders in investigating the effect of gut microbiota on toxin status. After adjusting for both factors, three species were identified that discriminated by toxin status, that is, Toxin+ and Toxin− groups. *A. muciniphila* had the strongest association with Toxin+ status, followed by *Flavonifractor plautii* and *Bifidobacterium adolescentis* after correcting for multiple comparisons via FDR adjustment (*q* < 0.25 as cut-off) (Fig. 5). Similar analysis of ARGs at the type-level revealed differences in relative abundance of several ARG types among individuals classified as Toxin− compared to Toxin+ status (Fig. S4).

In pairwise comparisons across all three toxin groups, no specific species consistently distinguished individuals in group 1 (Toxin+). However, individuals in group 3 (Toxin−/PCR−) showed elevated levels of *Streptococcus mutans* across all comparison groups (Fig. S5). Nevertheless, caution is advised in concluding these pairwise comparisons due to their limited sample size.

### Metabolic pathway-level comparison

**Fig 6:**
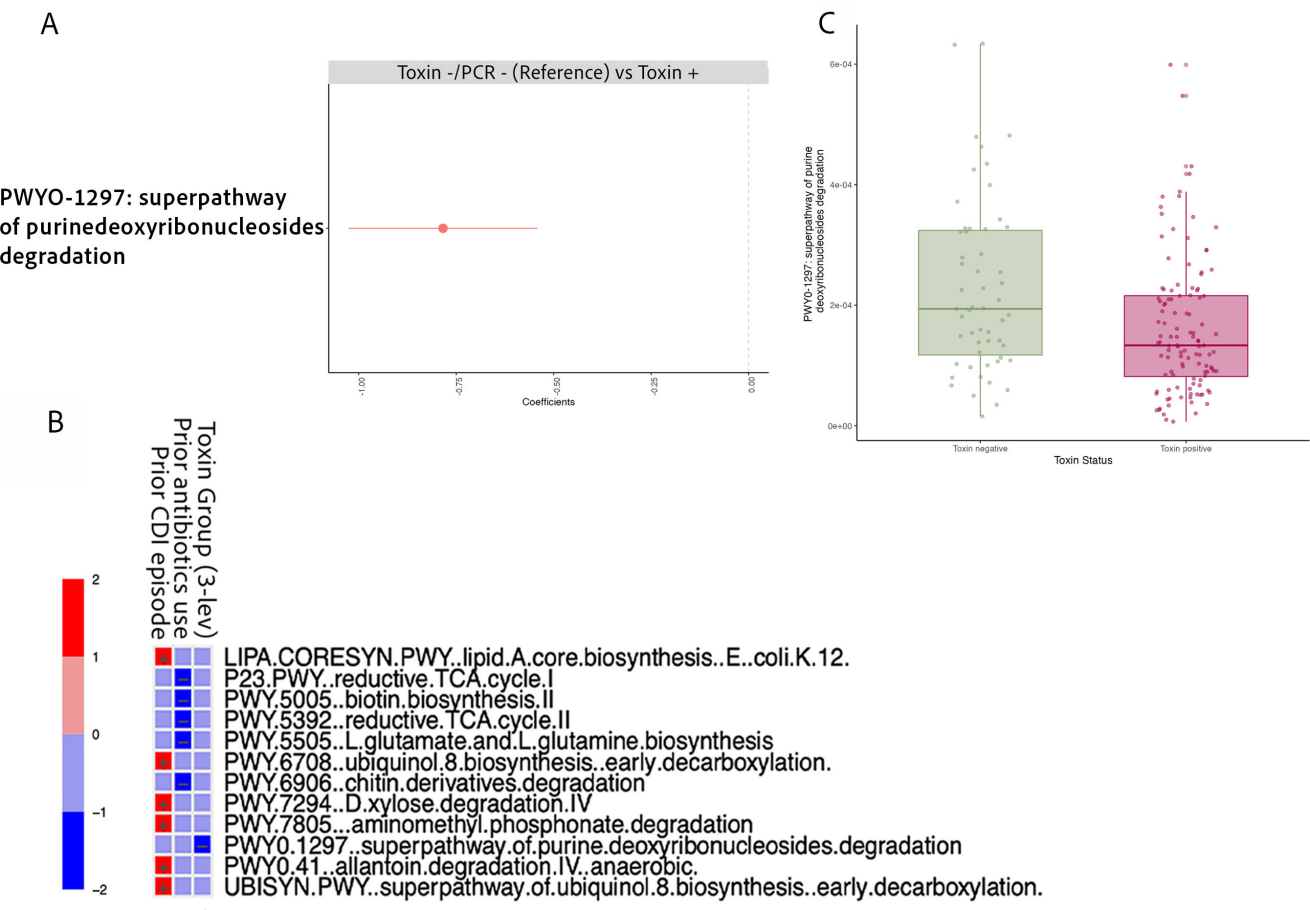
Differential metabolic pathway profiles associated with *C. difficile* toxin group or toxin status, adjusting for prior antibiotics exposure and previous CDI episode. Across all panels, only pathways with *q*-values <0.25 are shown. (**A**) MaAsLin2 results from pairwise comparisons of metabolic pathways between toxin groups. Only significant differences between Toxin−/PCR− and Toxin+ are shown (no significant differences observed for other comparisons). (**B**) Heatmap displaying associations between metabolic pathways and additional covariates included in the model (e.g., prior antibiotics exposure, previous CDI episode). (**C**) Boxplot with individual jittered points illustrating the relative abundance of metabolic pathways by toxin status (Toxin+ vs Toxin− groups) based on MaAsLin2 results.

We aimed to gain deeper insights into the microbiome signature differences across toxin groups by examining functional pathway profiles, beyond just taxonomic profiling. Our multivariable DA analysis adjusting for both prior antibiotics exposure and previous CDI episode revealed that the superpathway of purine deoxyribonucleosides degradation was enriched in Toxin−/PCR+ samples when compared to Toxin+ samples, making it the most distinguishing feature (Fig. 6A). From the same multivariable analysis, we show the relationship between individual pathways and covariates (Fig. 6B). This association remained consistent when analyzing by toxin status (Fig. 6C).

## DISCUSSION

Diarrheal symptoms, which often prompt initial diagnostic processes, are not specific to CDI, and often individuals who were thought to have CDI had an alternative etiology for diarrhea (36, 37). Our analysis provides a comprehensive description of antibiotic exposure, the stool microbiome, and *C. difficile* toxin production and gene presence. Throughout the study, we observed the influence of antibiotic exposure and specific key species on differential toxin production and gene presence. *Enterococcus* species were closely linked with prior antibiotics exposure, and *A. muciniphila* abundance distinguished participants with Toxin+ status after controlling for prior antibiotics exposure and a previous CDI episode. Moreover, we found that neither the abundance nor the detection of *C. difficile* alone is sufficient to discern true CDI cases. We identified key differences in the purine metabolism pathway that distinguish toxin groups, particularly in Toxin−/PCR− patients. Our analysis highlights the complexity of the microbiome in *C. difficile*-related diseases, and further research is needed to better understand the biomarkers related to toxin production and gene presence.

Ferretti et al. analyzed publicly available shotgun metagenomic sequencing data sets to identify microbiome signatures of CDI; their analyses indicate that the prevalence of *C. difficile* is frequently overestimated and reinforce concerns regarding overprescribing of antibiotics for suspected CDI (38). In our study, 35% of participants with Toxin− status were treated for “incident CDI” despite not meeting the criteria for true clinical CDI.

Across all our participants, 53% had prior antibiotics exposure. Among all antibiotic classes, vancomycin was the most frequently used, both as a prior exposure and as a first-line treatment for incident CDI (72% of the total population). Our data also revealed a significant enrichment of *Enterococcus* species among all individuals who were colonized (Toxin−/PCR+ and Toxin−/PCR−) or infected with *C. difficile* (Toxin+). Moreover, the abundance of *E. faecium* demonstrated a strong correlation with clusters of vancomycin-associated ARG subtypes. These findings align with previous studies indicating that enterococci thrive in antibiotic-perturbed microbiomes and may play a role in antibiotic-associated diarrhea (38, 39). A murine study by Smith and colleagues found that exposure to antibiotic-resistant *E. faecium* and *E. faecalis* resulted in increased toxin production by *C. difficile* (40). *C. difficile* can grow as biofilms within the mucus layer (41), and *E. faecalis* has been shown to exhibit a synergistic effect in biofilm formation with *C. difficile* (40, 42). Biofilms protect from antibiotics and immune responses and play a vital role in the survival and infection of *C. difficile* (43). Moreover, biofilms provide an environment conducive to horizontal gene transfer between *E. faecalis* and *C. difficil*e (40, 44). The transfer of *C. difficile* genes to enterococci may, in turn, support the expansion and fitness of antibiotic-resistant *E. faecalis* in the gut (40), underscoring concerns for vancomycin-resistant enterococci resistance.

Our data showed that the abundance of *A. muciniphila* was higher in individuals with true CDI (Toxin+ status) after controlling for prior antibiotics exposure and previous CDI episodes. *A. muciniphila* has previously been linked with a healthy gut microbiome (45) and has also been hypothesized to serve as a marker of microbiome dysbiosis associated with recurrent CDI (46). Data from a clinical trial of the microbiome restoration therapy RBX2660 showed that patients whose CDI did not recur after a single dose of RBX2660 had stable and lower (<25%) *A. muciniphila* abundance after 30 days. In contrast, participants who required repeat doses of RBX2660 had severely perturbed microbiota at days 0 and 7 and greater variation in *A. muciniphila* abundance; the relative abundance of *A. muciniphila* exceeded 40% in several samples (46). *C. difficile* cannot metabolize mucin glycans due to the absence of glycosyl hydrolase enzyme (41, 47). *A. muciniphila* is a mucin-degrading specialist with a broad range of glycosyl hydrolase enzymes that can degrade mucins that serve as a nutrient source, thereby facilitating the growth of *C. difficile* (41, 47). The role of *A. muciniphila* in CDI as either a surrogate marker or contributor to pathogenesis is an important area for further study.

The relative abundance of *F. plautii* and *B. adolescentis* was also higher in individuals with true CDI (Toxin+ status). *F. plautii* degrades flavonoids, which are important anti-inflammatory mediators; low levels of flavonoids may lead to higher levels of inflammation, which may be conducive to *C. difficile* (48). *B. adolescentis* has been described as a beneficial microbe and has been used in bacteriotherapy studies of CDI (49, 50). *F. plautii* and *B. adolescentis* are examples of taxa that may serve as critical members of the healthy gut microbiota and also contribute to poor outcomes in states of extreme dysbiosis. Alternatively, these associations may be spurious or related to probiotic use.

We identified one distinct metabolic pathway differentiating toxin groups and status, with purine degradation notably higher in the Toxin−/PCR− group than in the Toxin+ group. This specific pathway has been studied in *E. coli* due to their ability to utilize purine deoxyribonucleosides as carbon and energy sources (51). Purine metabolism pathways are prevalent among gut bacteria, as these pathways are an important source of essential nutrients (52). Studies suggest that purine degradation can foster beneficial interaction for both healthy commensals (52) and also for *C. difficile*, particularly through the utilization of uric acid (53). *C. difficile* can metabolize a large number of nutrients for growth, and specific subsets of metabolic pathways may be utilized that allow *C. difficile* to flourish in a range of dysbiotic microbial communities (54). Additional studies of metabolic pathways involved in *C. difficile* colonization and pathogenesis may facilitate the identification of biomarkers that can be used in diagnostics.

Our study had limitations. When three-level toxin groups are considered, caution is warranted in drawing definitive conclusions given the limited sample sizes in groups 2 and 3. The stool samples were collected at a single point in time, limiting our ability to establish temporality. As a result, we cannot determine whether the observed interaction between the gut microbiota and the spectrum of *C. difficile*-related disease manifestations is driven by the gut microbiota influencing *C. difficile* or if it is the impact of *C. difficile* on the gut microbiota. We did not include healthy controls; the goal of our study was to identify microbiome signatures related to toxin production and toxin gene presence. Thus, we restricted our studies to individuals who were colonized with *C. difficile* or experiencing true CDI. Lastly, specific taxa and metabolic pathway information cannot be linked directly. However, by conducting a statistically rigorous DA analysis at the species level using shotgun metagenomics and considering potential confounder considerations, we present sets of important species and their composite functional capabilities. This consolidated approach provides a broader understanding of associations between the gut microbiota and *C. difficile* toxin production and toxin gene presence. Future diagnostic and therapeutic strategies should consider the gut microbiome and clinical factors such as prior antibiotics exposure and previous CDI episodes in *C. difficile*-related disease manifestations.

## Data Availability

The deidentified data that support the findings of this study are available upon request from the corresponding author. The shotgun metagenomic sequencing reads have been deposited with the NCBI Sequence Read Archive and are available under BioProject PRJNA1056768.

## SUPPLEMENTAL MATERIAL

Supplemental Material msphere.00435-25-s0001.docx Methods S1, Figures S1-S5, and Tables S1-S4.

ASM does not own the copyrights to Supplemental Material that may be linked to, or accessed through, an article. The authors have granted ASM a non-exclusive, world-wide license to publish the Supplemental Material files. Please contact the corresponding author directly for reuse.
