# Supplementary material for: Microbiome signatures of Clostridioides difficile toxin production and toxin gene presence: a shotgun metagenomic approach
Source: mSphere. 2025 Sep 25;10(10):e00435-25. doi: 10.1128/msphere.00435-25 (PMC12570482; doi:10.1128/msphere.00435-25)
Supplement: Supplemental Material — Methods S1, Figures S1-S5, and Tables S1-S4. [file msphere.00435-25-s0001.docx]

Supplementary Materials for

**Microbiome signatures of *Clostridioides difficile* toxin production and toxin gene presence: A shotgun metagenomic approach**

Jiye Kwon^1,2^, Maria A. Correa^3^, Yong Kong^4,5^, William Pelletiers^1,6^, Martina Wade^1^, Danyel Olson^3^, Melinda M. Pettigrew^7^

Corresponding author: mpettigr@umn.edu

**The PDF file includes:**

Methods S1

Figs. S1 to S5

Tables S1 to S4

**Supplementary methods**

S1. Differential abundance analysis using MaAsLin2

We performed differential abundance analysis using the Microbiome Multivariable Association with Linear Models 2 (MaAsLin2) package in R across the three datasets: 1) taxonomic data (species-level, relative abundance), 2) antibiotic resistance genes (type-level), and 3) metabolic pathway abundance. Both univariate and multivariable models were conducted. In univariate models, we included four separate fixed effects independently --- toxin groups (3-level), toxin status (Toxin + vs Toxin $\boldsymbol{-}$), prior antibiotics exposure (yes/no), and previous CDI episode (yes/no). For multivariable models, the primary outcome variable was either toxin group or toxin status, concurrently including other covariates (prior antibiotics exposure and previous CDI episode) as fixed effects to both analyses.

Analyses used log transformation, no normalization, and filtering thresholds of 0.001 for minimum feature variance and 0.01 for minimum relative abundance. For metabolic pathway data, we applied a threshold of 0.00001 due to overall lower signal.

A representative example of the R code implementation is as follows:

Univariate analyses:

Maaslin2:: Maaslin2(input_data = input_taxa.rev,

input_metadata = all.meta,

output = './250511_univar_threeEXPGroup',

fixed_effects = c("toxin_groups "),

transform="LOG",

normalization = "NONE",

min_variance = 0.001,

min_abundance = 0.01)

Multivariable analyses:

Maaslin2:: Maaslin2(input_data = input_taxa.rev,

input_metadata = all.meta,

output = './250511_multivar_threeEXPGroup',

fixed_effects = c(“toxin_groups”, “Prior_antibiotics_exposure”, “Prev_CDI_episode”),

transform="LOG",

normalization = "NONE",

min_variance = 0.001,

min_abundance = 0.01)

**Supplemental Table 1. Univariate logistic regression results associated with toxin status.** Toxin+ vs Toxin- i.e., Group 1 vs Groups 2 & 3 combined.

| **Antibiotic Class** | **Toxin +**  **(n = 117)**** | **Toxin –**  **(n = 55)**** | **OR (95% CI)** | ***P* Value** |
| --- | --- | --- | --- | --- |
| Any prior antibiotics | 71 | 21 | 2.50 (1.3 - 4.9) | 0.006 |
| Beta-lactam | 53 | 8 | 4.86 (2.2 – 11.9) | < 0.001 |
| Cephalosporin | 36 | 4 | 5.67 (2.1 – 19.8) | 0.002 |
| Fluoroquinolone | 21 | 1 | 11.81 (2.4 – 214.7) | 0.02 |
| Vancomycin | 27 | 7 | 2.05 (0.9 – 5.4) | 0.12 |

* OR = odds ratio; exp(beta)

* Toxin Status

** The total column sum may exceed the total number of patients in each toxin status, as an individual may have had exposure to more than one type of antibiotic class.

**Supplemental Table 2. Multivariable PERMANOVA model summary and results.** Columns include model terms, PERMANOVA P-values, and betadisper P-values. The betadisper test evaluates the assumption of homogeneity of group dispersions (i.e., variances) across groups.

| **Terms** | **PERMANOVA**  R function: adonis2 | **multivariate homogeneity of group dispersions (variances)**  (R function: betadisper) |
| --- | --- | --- |
| Toxin groups | p = 0.22 | p = 0.33 |
| Case definition | p <0.001 | p <0.001 |
| Prior antibiotics exposure | p = 0.004 | p = 0.007 |
| Prior antibiotics exposure* Toxin groups | P = 0.65 |  |

**Supplemental Table 3. Pairwise comparison of beta-diversity among toxin groups.** Values shown below are false discovery rate (FDR) adjusted P-values correcting for multiple comparison.

|  | Toxin + (n = 116) | Toxin -/PCR+ (n = 22) |
| --- | --- | --- |
| Toxin -/PCR+ (n = 22) | 0.80 | - |
| Toxin -/PCR- (n = 33) | 0.72 | 0.72 |

# Supplemental Figures

**Supplemental Figure 1. Correlation plot of key microbial species across all study participants.** Figure displays pairwise correlations of species-level relative abundances for taxa with >60% prevalence among study participants. Only statistically significant correlations are shown, with color representing the strength and direction of association.


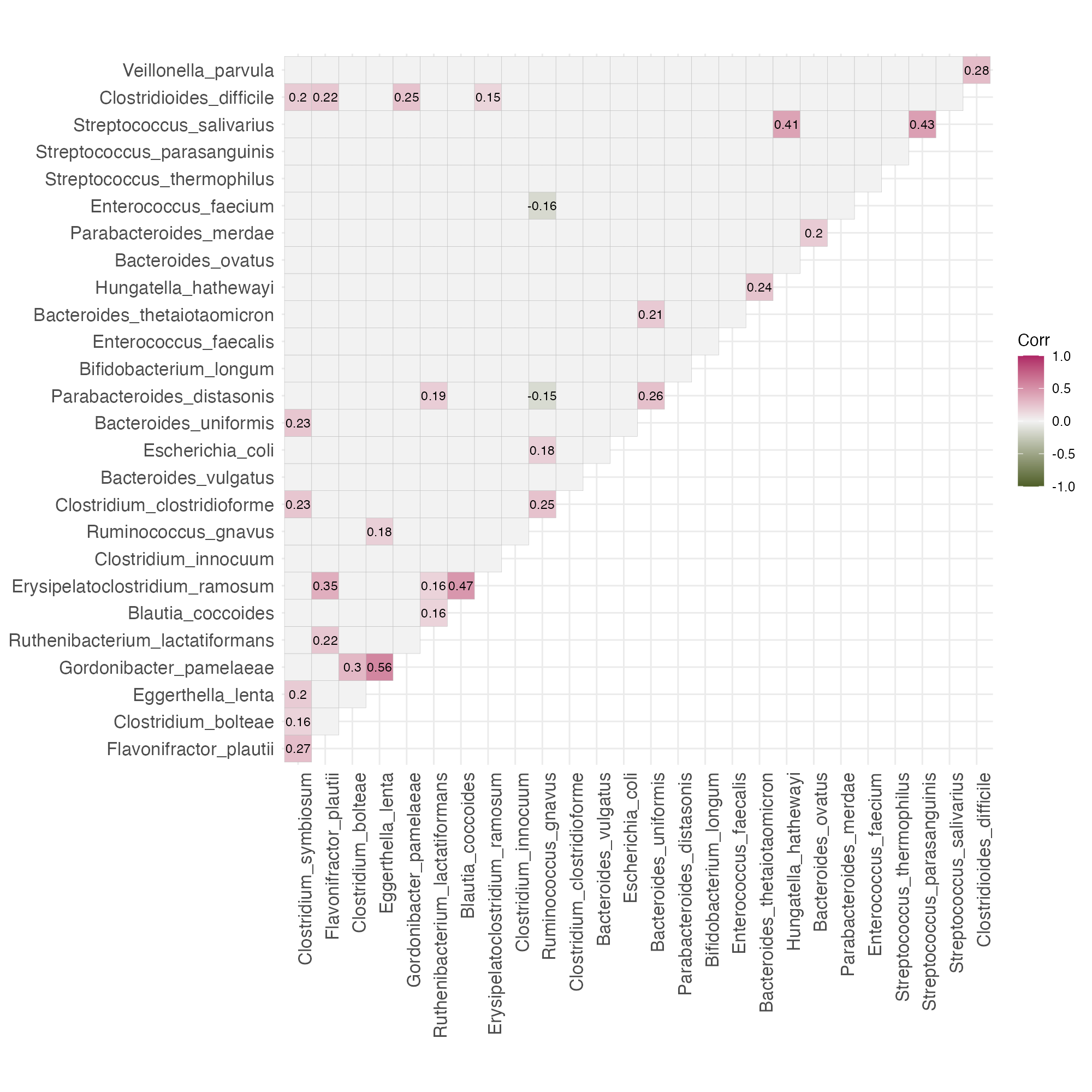


**Supplemental Figure 2. Comparison of antibiotics resistance gene (ARG) types by prior antibiotics exposure.**


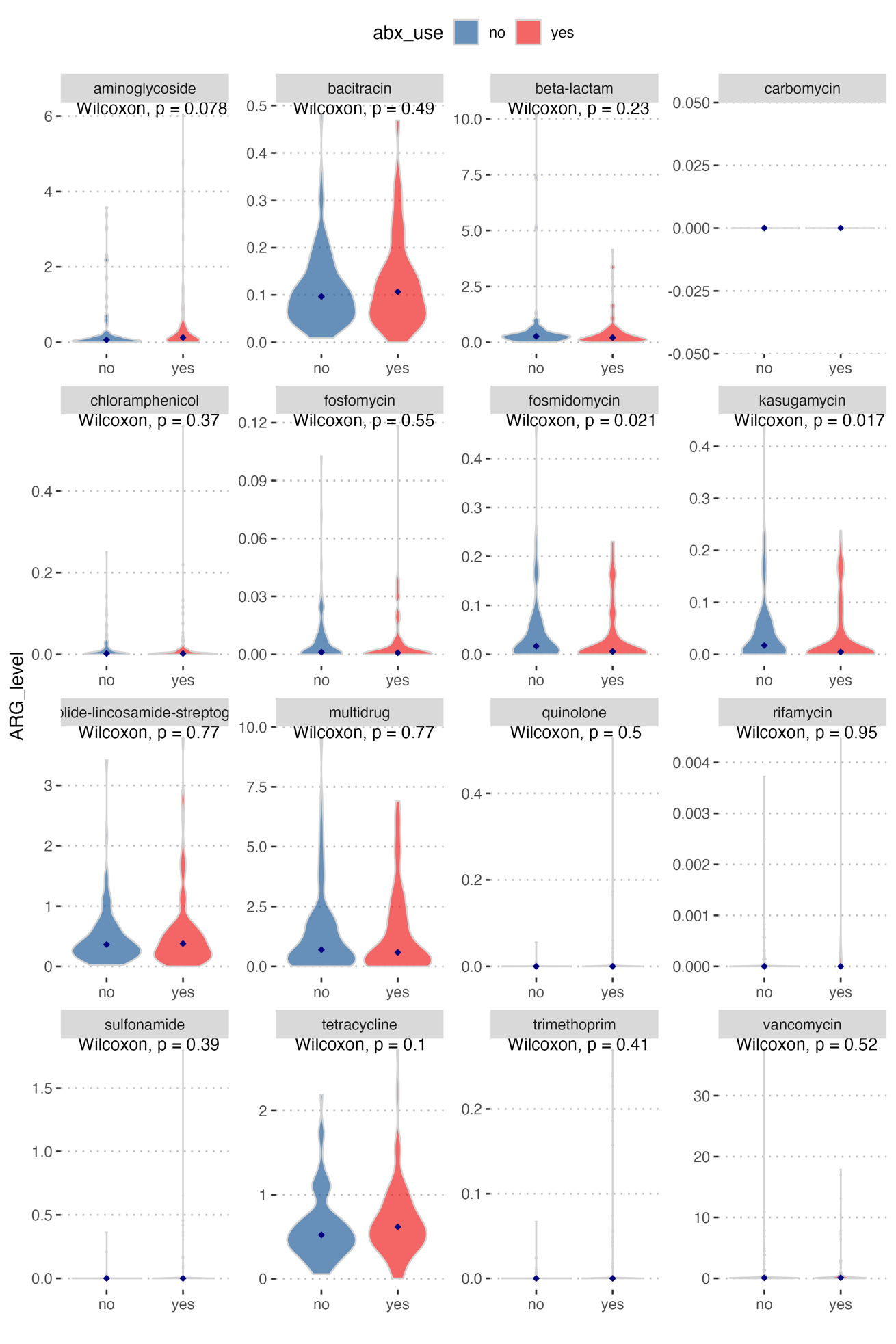


**Supplemental Figure 3. Significant microbial taxa associated with previous CDI episode.**

Taxa with positive coefficients are more abundant in individuals with a history of CDI. Colors indicate taxonomic Family of the microbial feature, and the size of circle corresponds to magnitude of statistical significance. Features with q-value of <0.25 were plotted.


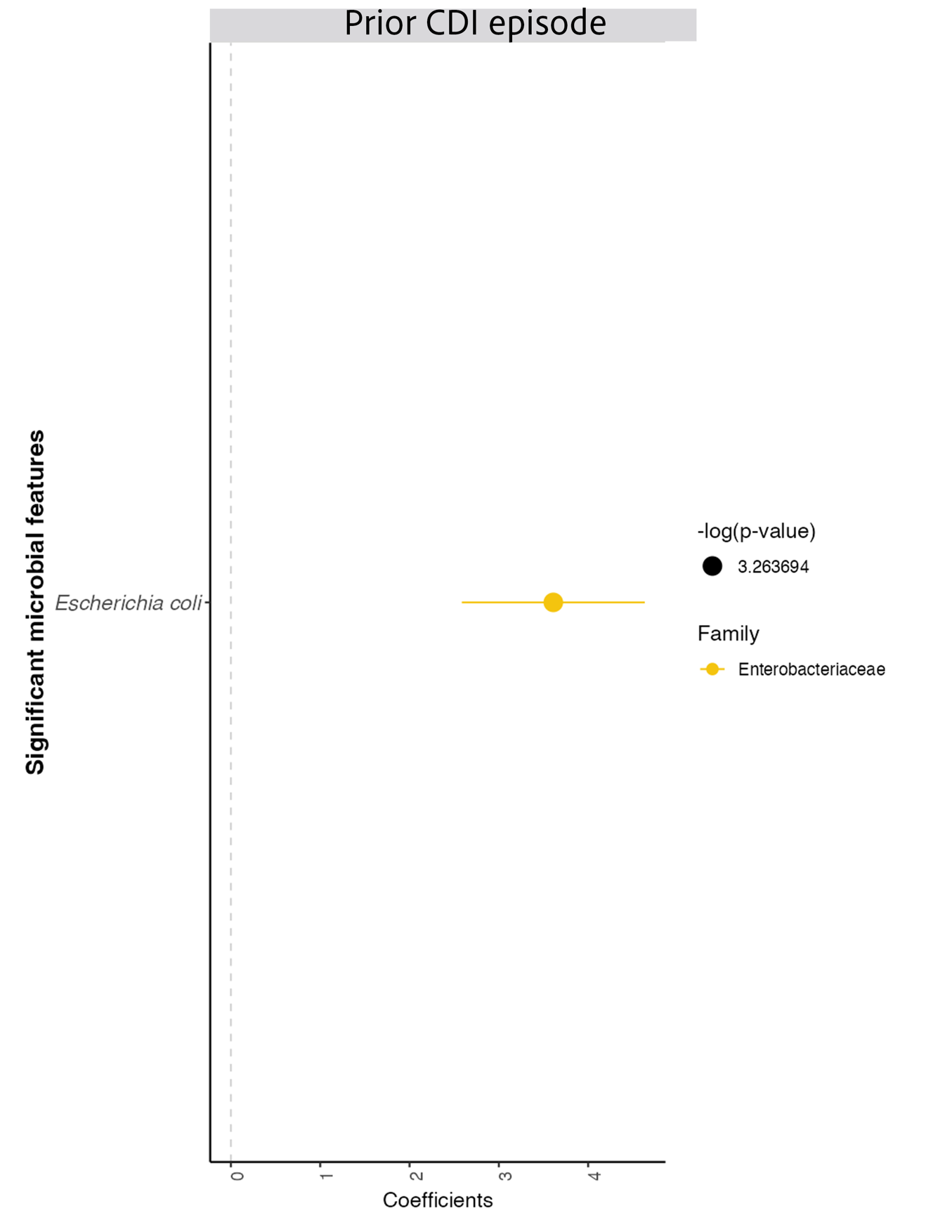


**Supplemental Figure 4. Significant antibiotics resistance gene (ARG) types with different toxin status, adjusted for prior antibiotics exposure and previous CDI episodes.** Colored circles indicate type-level ARG more abundant in Toxin $-$ individuals compared to Toxin+. Circle sizes correspond to the magnitude of statistical significance. Only features with a q-value < 0.25 are plotted.


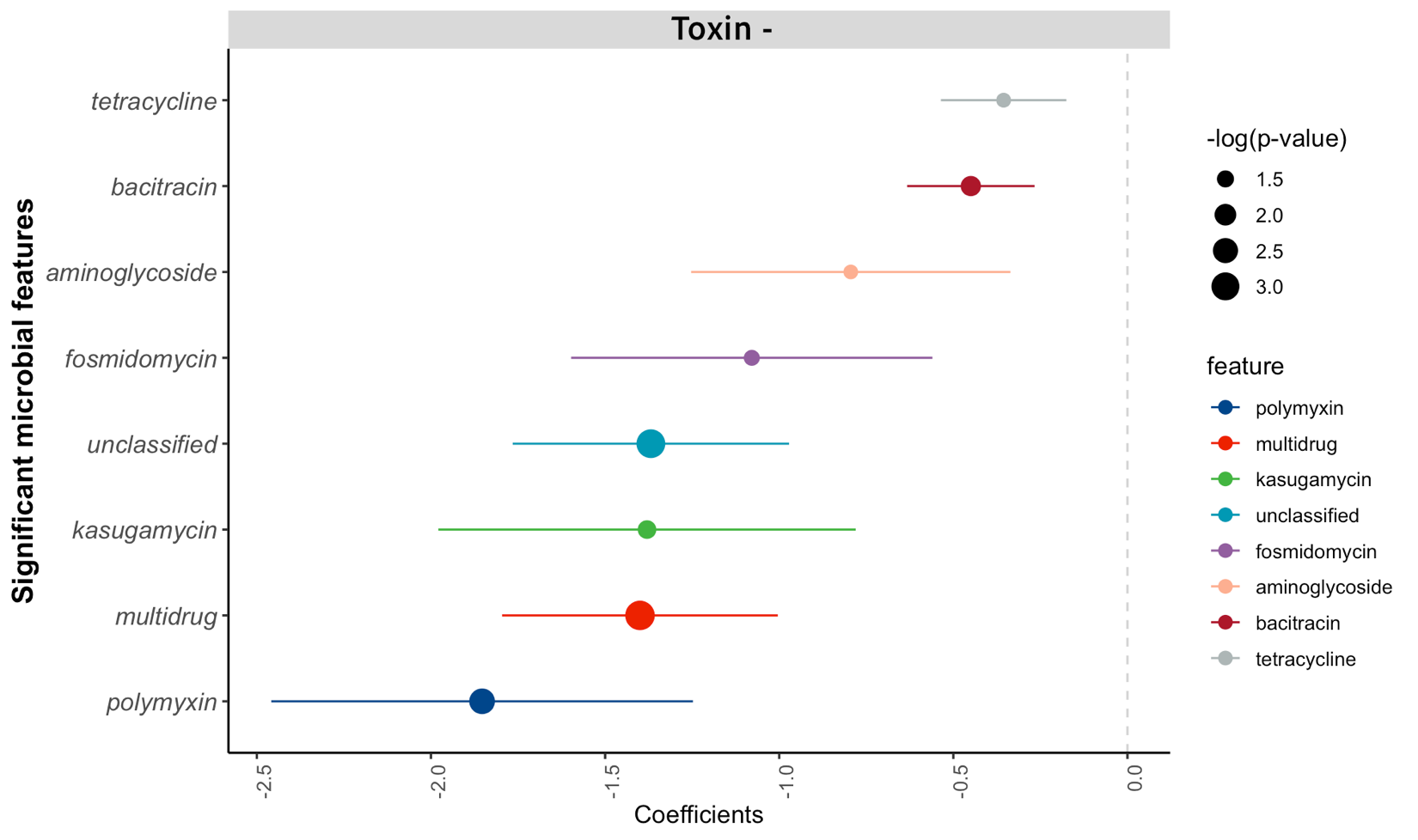


**
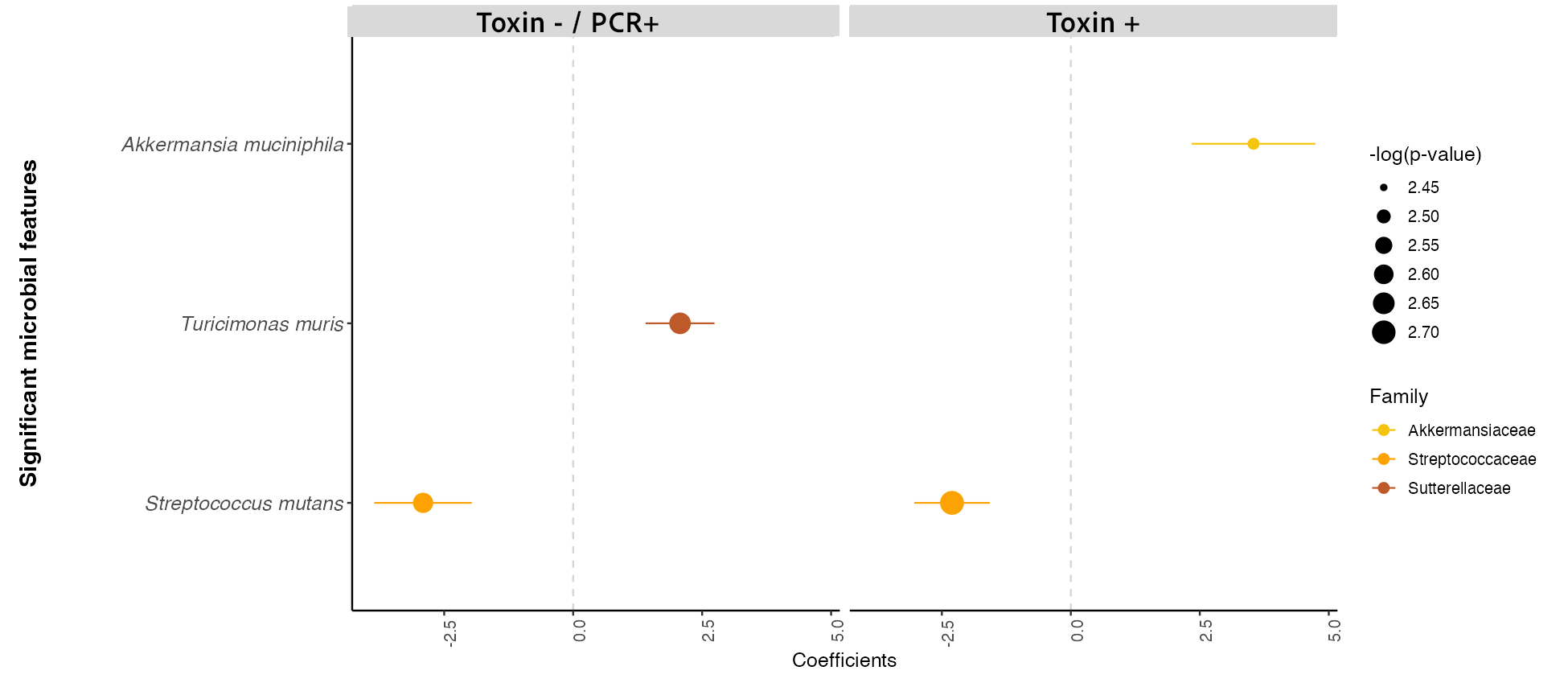
**

**Supplemental Figure 5. Significant microbial taxa associated with different toxin groups, adjusted for prior antibiotic exposure and previous CDI episodes.** Pairwise comparisons are shown, with the reference group Toxin-/PCR- (Group 3) and the comparison group shown in the panel title. Taxa with positive coefficients are more abundant in individuals indicated in the panel title, and negative coefficients are more abundant in Toxin-/PCR- (Reference). Colored circles indicate the taxonomic Family of each microbial feature, with circle size corresponding to the magnitude of statistical significance. Only features with a q-value < 0.25 are plotted. Comparisons are shown only for groups with at least one significant microbial feature.

**Supplemental References**

1. M. J. Anderson, A new method for non‐parametric multivariate analysis of variance. *Austral Ecology*

**26**, 32-46 (2001).

2. Oksanen J, Blanchet FG, Friendly M, Kindt R, Legendre P, McGlinn D, Minchin PR, O'Hara RB,

Simpson GL, Solymos P, Stevens MHH, Szoecs E, Wagner H. 2019. vegan: Community Ecology

Package, <https://CRAN.R-project.org/package=vegan>.
